# Editorial: Modulation of Human Immune Parameters by Anticancer Therapies

**DOI:** 10.3389/fimmu.2020.621556

**Published:** 2020-12-02

**Authors:** Ulrich Sack, Attila Tarnok, Frank Preijers, Ulrike Köhl, Il-Kang Na

**Affiliations:** ^1^ Medical Faculty, Institute of Clinical Immunology, Leipzig University, Leipzig, Germany; ^2^ Fraunhofer Institute for Cell Therapy and Immunology (IZI), Leipzig, Germany; ^3^ Institute for Medical Informatics, Statistics and Epidemiology (IMISE), University of Leipzig, Leipzig, Germany; ^4^ Department Precision Instruments, Tsinghua University, Beijing, China; ^5^ Radboud University Nijmegen Medical Centre, Nijmegen, Netherlands; ^6^ Institute for Cellular Therapeutics, Hannover Medical School, Hannover, Germany; ^7^ Department of Hematology and Oncology, Charité – Universitätsmedizin Berlin, Corporate Member of Freie Universität Berlin, Humboldt-Universität zu Berlin, and Berlin Institute of Health, Berlin, Germany; ^8^ Experimental and Clinical Research Center (ECRC), Berlin, Germany; ^9^ Berlin Institute of Health (BIH), Berlin, Germany; ^10^ German Cancer Consortium (DKTK), partner site Berlin, Heidelberg, Germany

**Keywords:** immunoncology, flow cytometry, checkpoint inhibition/blockade, immune modulation, tumor-immune cell interaction

Immunoncology is among the most important hallmarks of immunotherapy revolution of cancer medicine. Here, we compiled reviews and original research articles reflecting current developments in immunoncology.

Novel therapies modulate the complex interaction between tumor and immune system (**Figure 1**). Multiparametric flow cytometry (FCM)**** is a key analytical tool contributing over 1,000 research articles/year to the field. As a quantitative single-cell technology, FCM reliably and reproducibly identifies rare populations, detects subtle changes in modulatory signals, and assesses time-sensitive antigenic expression patterns. State-of-the-art equipment, fast sophisticated software, and flexibly labeled monoclonal antibodies allow rapid analyses with high sensitivity and specificity, even in routine applications. Lambert et al. explain how new analytes are added to the portfolio of diagnostic and research laboratories. Sample preparation, antibody titration, and appropriate controls are central in cytometric analysis and must be controlled with the necessary rigor and reproducibility (1).

**Figure 1:**
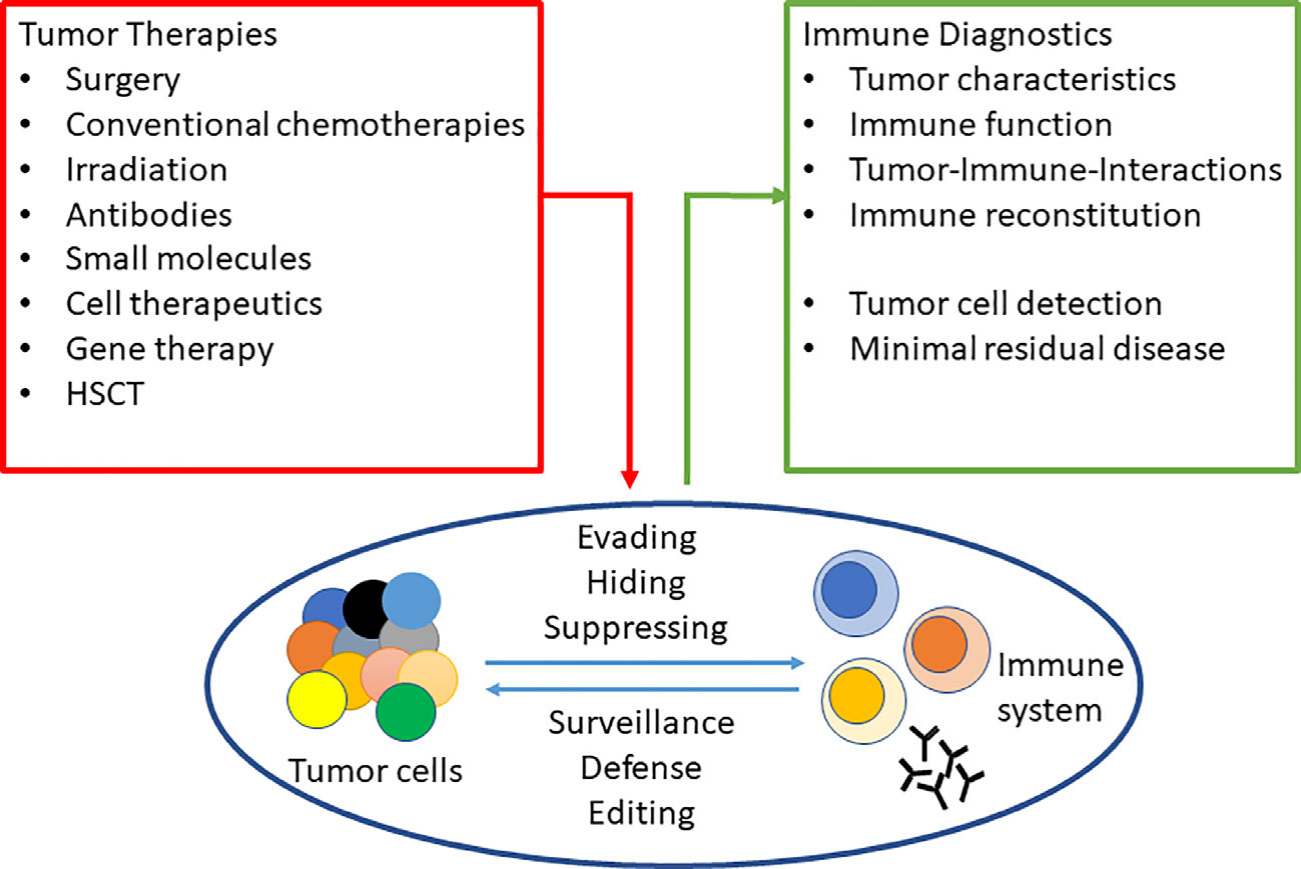
Tumor-immune-interactions and investigation by flow cytometry.

Although tumor cell**** analysis is a key application of cytometry (2, 3), this research topic is dedicated to the modulation of immune parameters, and we only included work focusing on ****tumor-immune-cell interaction**** and its disease-course impact.

Dendritic cells (DCs) are crucial in tumor protection (4). Lu et al. dissect the interaction of DCs with non-small cell lung cancer (NSCLC) cells, which can induce an immunosuppressive microenvironment and evade immune surveillance. Analysis of costimulatory molecules and pro-/anti-inflammatory cytokines reveals new subpopulations of CD1c+ DCs in coculture with NSCLC. Particularly, the expression of signal molecules and pro-inflammatory cytokines are suppressed, whereas the secretion of anti-inflammatory cytokines by DCs is upregulated, suggesting that NSCLC can induce tolerogenic DCs, blocking DC-mediated anti-tumor immunity.

Chemokines and their corresponding receptors play a pivotal role in orchestrating trafficking of immune cells to fulfill their next tasks. CXCL10 has been associated with T cell recruitment into tumors. Meng et al. link an increased CXCL10 expression and T cell infiltration with autophagy inhibition in gastric cancer (GC). Since autophagy was associated with GC cell survival and therapy resistance, autophagy inhibition is considered a potential GC treatment strategy, which might also favorably effect T cell recruitment into the tumor.

Inflammation is central in tumorigenesis underlining close interactions between immune system and tumor. Besides adaptive immunity, innate immunity is crucial in tumor defense (5). Stein et al. address the role of the inflammatory cytokine TNF-α in colorectal cancer (CRC). CRC has commonly good prognosis, if detected early. With distant metastasis, 5-year survival rate drops below 10% with little therapeutic progress. The metastasis-associated oncogene in CRC 1 (MACC1) is involved in CRC metastasis, induces cell proliferation and motility, supports cell survival, and redirects metabolism. Also, in several other solid cancers, MACC1 is a potential target for late forms of metastasis. The authors demonstrate that TNF-α triggers upregulation of MACC1 mRNA and protein *via* induction of c-Jun expression, resulting in promoted CRC-cell migration. MACC1 induction was successfully inhibited by MACC1 and c-Jun knockdown as well as anti–TNF-α and anti-TNFR1 blocking antibodies, providing potential therapeutic targets for treating inflammation-associated CRC.

Tumor-immune-cell interactions are decisive in the disease course but not yet fully understood and addressed by Plesca et al. In various cancers, high densities of CD45RO+ T-helper1 cells and CD8+ T cells are associated with improved outcome, M2 macrophages rather with worsened prognosis (5). This can also be applied to the expected response to anti-programmed cell-death-protein 1 (PD-1). Anti–PD-1 therapy affects an increased density of tumor-infiltrating T cells in responders, and increased frequency of melanoma-infiltrating TCF7+CD8+ T cells. However, tumor-infiltrating PD-1+CD38hi CD8+ T-cells are associated with anti–PD-1 resistance that favors implementation of immunoprofiling before checkpoint inhibition therapy.

Based on the manifold relationships between immune system and tumor, the numerous parallels between immunopathology and tumor therapy are not surprising. Khoy et al. present Natalizumab in multiple sclerosis (MS) as a typical example for immune therapies and precursor of today’s antibody therapies for tumors. MS is a chronic demyelinating disease of the CNS with an autoimmune component. Among the recently available disease-modifying therapies, Natalizumab, a monoclonal antibody against VLA-4 integrin, effectively inhibits cell migration to tissues including the CNS, thereby inhibiting disease progression. Since also immune function is impaired, immunomonitoring during therapy is important to detect adverse effects.

Klöß et al. report on challenging examples that bridge between treatment of cancer and immune-mediated diseases, major hurdles are suitable experimental models reflecting the complex tumor-immune-interactions during treatment for identifying new therapies. In addition to patient-derived tumor xenotransplants (PDX) (humanized) mouse models, *ex vivo* approaches to cancer modeling like microfluidic human organs-on-chips are shown. Better understanding of treatment mechanisms and side effects permitted the development of novel targeted cell-, drug-, and biological-based therapies. Progress of our knowledge about inhibitory and stimulatory immune mechanisms associated with autoimmune diseases enable novel strategies to tackle autoimmunity using regulatory CAR-T cells (CAR Treg) of natural killer cells (NK) (6, 7).

Cell-based therapies, particularly CAR-T or CAR-NK cells redirected against aggressive leukemia and lymphoma, have taught lessons to improve immunoncology. Identification of tumor-associated antigens and the respective target-to-effector interaction and understanding how to overcome the immunosuppressive tumor-microenvironment are #1 challenges as addressed in previous Frontiers in Immunology (8, 9). Current development in CAR-NK cells for leukemia treatment (10–12) must be applied also to solid tumors.

Gibellini et al. review single cell approaches to profile the response to immune checkpoint inhibitors. Since tumor cells are highly variable, single-cell analysis like polychromatic FCM, single-cell sequencing, or high-resolution imaging can be employed to examine rare tumor cells. These methods allow analyses in unprecedented detail, fostering understanding of molecular and cellular interactions between cancer and the immune system.

Unfortunately, analysis of tumor cells and immune signatures is not per-se successful. For many cancer types, finding cancer stem cells (CSCs) is essential for therapy optimization as Walcher et al. highlight. They review the most used CSC markers focusing on lung, gastric, liver, breast, and colon cancer and myeloid leukemias. CSCs are an integer part of tumors, drive tumor initiation and can cause relapses. To date, several biomarkers characterizing CSCs have been identified and correlated with diagnosis, therapy, and prognosis. However, CSCs have a high plasticity altering their phenotypic and functional appearance. Such changes are induced by chemo- and radiotherapy as well as by senescent tumor cells, modifying the tumor microenvironment. One source of CSCs is ****circulating tumor cells that are not part of this issue but are addressed in recent overviews (13, 14).

The last article reports on ****drug actions immunomonitored by high-content FCM (15, 16). Parry et al. investigated long-term Ibrutinib therapy in B-Cell Chronic Lymphocytic Leukemia (CLL). CLL is associated with immunosuppression and susceptibility to infection. Investigating virus-specific CD8+ T cells, authors could demonstrate a reduction in PD-1 expression and increased cytokine production following stimulation. The results suggest that Ibrutinib therapy is associated with recovery of pathogen-specific T cells in B-CLL thus contributing to reduced risk of infection.

In summary, we hope that this research topic adds important facets to the picture of immunoncology.

## Author Contributions

US, AT, FP, and IN developed the topic, identified the authors, supported the publication process, and wrote this editorial. UK gave advice, supported selection of authors, and co-edited this editorial. All authors contributed to the article and approved the submitted version.

## Conflict of Interest

The authors declare that the research was conducted in the absence of any commercial or financial relationships that could be construed as a potential conflict of interest.

## References

[1] Laskowski TJ Hazen AL Collazo RS Haviland D Rigor and Reproducibility of Cytometry Practices for Immuno-Oncology: A multifaceted challenge. Cytometry A (2020) 97:116–25. 10.1002/cyto.a.23882 31454153

[2] Ijsselsteijn ME Brouwer TP Abdulrahman Z Reidy E Ramalheiro A Heeren AM Cancer immunophenotyping by seven-colour multispectral imaging without tyramide signal amplification. J Pathol Clin Res (2019) 5:3–11. 10.1002/cjp2.113 30191683 PMC6317065

[3] Frolich S Robker R Russell D Development of Automated Microscopy-Assisted High-Content Multiparametric Assays for Cell Cycle Staging and Foci Quantitation. Cytometry A (2020) 97:378–93. 10.1002/cyto.a.23988 32083400

[4] Huang Y Wang Y Chang Y Yuan X Hao L Shi H Myeloid Neoplasms with Elevated Plasmacytoid Dendritic Cell Differentiation Reflect the Maturation Process of Dendritic Cells. Cytometry A (2020) 97:61–9. 10.1002/cyto.a.23953 31876105

[5] Arnaud-Sampaio VF Rabelo ILA Bento CA Glaser T Bezerra J Coutinho-Silva R Using Cytometry for Investigation of Purinergic Signaling in Tumor-Associated Macrophages. Cytometry A (2020) 97(11):1109–26. 10.1002/cyto.a.24035 32633884

[6] Kloess S Kretschmer A Stahl L Fricke S Koehl U CAR-Expressing Natural Killer Cells for Cancer Retargeting. Transfus Med Hemother (2019) 46:4–13. 10.1159/000495771 31244577 PMC6558329

[7] Kohl U Arsenieva S Holzinger A Abken H CAR T Cells in Trials: Recent Achievements and Challenges that Remain in the Production of Modified T Cells for Clinical Applications. Hum Gene Ther (2018) 29:559–68. 10.1089/hum.2017.254 29620951

[8] Hofer E Koehl U Natural Killer Cell-Based Cancer Immunotherapies: From Immune Evasion to Promising Targeted Cellular Therapies. Front Immunol (2017) 8:745. 10.3389/fimmu.2017.00745 28747910 PMC5506076

[9] Koehl U Toubert A Pittari G Editorial: Tailoring NK Cell Receptor-Ligand Interactions: An Art in Evolution. Front Immunol (2018) 9:351. 10.3389/fimmu.2018.00351 29535727 PMC5835124

[10] Kloss S Oberschmidt O Morgan M Dahlke J Arseniev L Huppert V Optimization of Human NK Cell Manufacturing: Fully Automated Separation, Improved Ex Vivo Expansion Using IL-21 with Autologous Feeder Cells, and Generation of Anti-CD123-CAR-Expressing Effector Cells. Hum Gene Ther (2017) 28:897–913. 10.1089/hum.2017.157 28810809

[11] Kloess S Oberschmidt O Dahlke J Vu XK Neudoerfl C Kloos A Preclinical Assessment of Suitable Natural Killer Cell Sources for Chimeric Antigen Receptor Natural Killer-Based “Off-the-Shelf”. Acute Myeloid Leukemia Immunother Hum Gene Ther (2019) 30:381–401. 10.1089/hum.2018.247 30734584

[12] Muller S Bexte T Gebel V Kalensee F Stolzenberg E Hartmann J High Cytotoxic Efficiency of Lentivirally and Alpharetrovirally Engineered CD19-Specific Chimeric Antigen Receptor Natural Killer Cells Against Acute Lymphoblastic Leukemia. Front Immunol (2019) 10:3123. 10.3389/fimmu.2019.03123 32117200 PMC7025537

[13] Xu M Zhao H Chen J Liu W Li E Wang Q An Integrated Microfluidic Chip and Its Clinical Application for Circulating Tumor Cell Isolation and Single-Cell Analysis. Cytometry A (2020) 97:46–53. 10.1002/cyto.a.23902 31595638

[14] Yu Q Yao Y Zhu X Gao Y Chen Y Wang R In Vivo Flow Cytometric Evaluation of Circulating Metastatic Pancreatic Tumor Cells after High-Intensity Focused Ultrasound Therapy. Cytometry A (2020) 97(9):900–8. 10.1002/cyto.a.24014 PMC7540359 32307867

[15] Li R Attari A Prytyskach M Garlin MA Weissleder R Miller MA Single-Cell Intravital Microscopy of Trastuzumab Quantifies Heterogeneous in vivo Kinetics. Cytometry A (2020) 97:528–39. 10.1002/cyto.a.23872 PMC7028488 31423731

[16] Zhang J Sun L Cui J Wang J Liu X Aung TN Yiqi Chutan Tang Reduces Gefitinib-Induced Drug Resistance in Non-Small-Cell Lung Cancer by Targeting Apoptosis and Autophagy. Cytometry A (2020) 97:70–7. 10.1002/cyto.a.23869 PMC7004076 31411813

